# Latent class analysis of occupational accidents patterns among Iranian industry workers

**DOI:** 10.1038/s41598-022-11498-w

**Published:** 2022-05-07

**Authors:** Behzad Saranjam, Islam Shirinzadeh, Kobra Davoudi, Zahra Moammeri, Amin Babaei-Pouya, Abbas Abbasi-Ghahramanloo

**Affiliations:** 1 Department of Occupational Health Engineering, School of Health, Ardabil University of Medical Sciences, Ardabil, Iran; 2 Health Department, Ardabil University of Medical Sciences, Ardabil, Iran; 3 Students Research Committee, School of Health, Ardabil University of Medical Sciences, Ardabil, Iran; 4 Department of Public Health, School of Health, Ardabil University of Medical Sciences, Ardabil, Iran

**Keywords:** Environmental sciences, Health occupations

## Abstract

Occupational accidents (OA) are among the main causes of disabilities and death in developing and developed countries. The aims of this study were to identify the subgroups of OA and assess the independent role of demographic characteristics on the membership of participants in each latent class. This cross-sectional study was performed on 290 workers between 2011 and 2017. Data gathering was done using the reports of accidents recorded in filed lawsuits. Descriptive statistical analysis was done using SPSS 16 and LCA was done using PROC LCA in SAS9.2. For latent classes were identified; namely “critical due to distractions and lack of supervision” (40.1%), “critical due to lack of safety knowledge” (27.9%), “critical due to fatigue and lack of supervision” (13.1%), and “catastrophic” (18.8%). After adjusting for other studied covariates, being illiterate significantly increased the odds of membership in “critical due to fatigue and lack of supervision” (OR = 4.05) and “catastrophic” (OR = 18.99) classes compared to “critical due to distractions and lack of supervision” class. Results of this study showed that the majority of workers fell under the latent class of critical due to distractions and lack of supervision. In addition, it should be noted that although a relatively small percentage of the workers are in the catastrophic class, the probability of occurring death is quite high in this class. Focusing on the education of workers and enhancing manager’s supervision and employing educated workers could help in reducing severe and catastrophic OA.

## Introduction

Occupational accidents (OA) are a major issue in the workplace around the world, including in Iran. Despite the fact that several definitions of OA have been developed, the exact meaning is represented in different sentences in the definitions. An OA is defined by the International Labor Organization as "an unanticipated and unplanned event that results in specific damage or injury”^1,2^.

Along with scientific advances and technological innovation, general welfare has increased in human societies. Every year, millions of OA happen in the world, which causes injuries and economic losses. OA is among the main causes of disabilities and death in developing or developed countries^3,4^.

There have been several initiatives to decrease the prevalence of OA; however, it is still catastrophically high. According to the World Health Organization (WHO), OA has still considered a health epidemic^5,6^. According to International Labor Organization (ILO), the developing countries are home to 60% of the world workforce, while only 5–15% of this population has access to occupational health services^7^. In addition, according to international organizations’ reports, every year two million lethal accidents happen in the world and 268 million injury-causing accidents happen in work and industrial environments. These estimates indicate that the mere economic consequences of occupational accidents are about 4% of the gross national product of developed countries^8,9^.

Identifying the causes of and factors in accidents is an essential step to prevent such accidents. One of the key tools to preventing industrial accidents is the descriptive-analytical examination of the accidents, which is performed to achieve a proper perception of these factors. Researchers from a variety of disciplines have tried to elaborate on the types of accidents and the factors. Along with uncovering the causes of such accidents, such studies describe and analyze OA and lead to understanding and predicting the accidents^10^. Considerable sums of money are spent in Iran every year to compensate for injuries and lost income of OA victims; this also affects the active workforce available in the country^3^. Amiri et al. analyzed OA with high risk in construction works and showed that head, face, and neck injuries had the highest frequency compared to other accidents^11,12^. The European Agency for Work Safety and Health estimated that 4.6 million OA happen in Europe every year, which means losing 146 million work hours. According to the agency, it is possible to motivate managers and employers to prevent such accidents by highlighting the financial damages of such accidents^13^. In 2002, 15 Americans lost their lives at work every day on average and 20% of the deaths were in the construction industry^10^. The descending trend of work accidents in the developed countries is undeniable, which indicates that it is possible to decrease the trend in other countries using a set of efficient programming and preparation^7^.

In order to obtain reliable models concerning a specific aspect of OA, an advanced data mining analysis should be carried out on a set of detailed data, in which a data unit refers to a single object of observation. One of the methods that can be used for this purpose is latent class analysis (LCA). LCA is an approach to identifying latent subgroups or classes among participants of a study^14^. This person-centered approach uses some indicator variables to identify latent classes. The underlying basis of the information of these subgroups is the existing similarities regarding indicator variables.

There is little information about OA in Iran. Although there are registered data about these accidents in Iran, however, there is no information about subgroups of these accidents in Iran. Based on the above-mentioned background, the aims of this study were to identify the subgroups of OA and assess the independent role of demographic characteristics on the membership of participants in each latent class after adjusting for other covariates.

## Materials and methods

### Study population and sampling framework

This cross-sectional study was carried out on OA recorded between 2011 and 2017. Information gathering was done using accident reports in filed lawsuits. Information was collected from studying the completed incident report form. These forms are the report form of accidents caused by the work of the Iranian Labor Office. The inclusion criteria were at least one year of work record, no physical impairment, and no chronic disease. Accidents cases with incomplete information were excluded.

The variables under study were age, marital status, education, shift work (morning, evening, night); individual causes of the accident (lack of skill and experience, fatigue and excessive sleepiness, distraction, and lack of safety knowledge); managerial causes of the accident (lack of adequate and accurate supervision, wrong order, lack of occupational safety and health training); severity of accident (death, temporary debilitation, and permanent debilitation); and injured member (the eyes, head, face, neck, waist, arm, forearm, wrist, hand fingers, feet, knees, and toes).

### Statistical analysis

Descriptive statistics were used to investigate the characteristics of workers and OA type, reason, severity, and distribution. Then, LCA was performed six times, using one to six classes to identify the best model that can fit the data. To find the best model, each candidate model was fitted 20 times with different starting values. To choose the final model, a few indices were calculated and compared across six models. These indices were likelihood-ratio statistics G2, Akaike information criteria (AIC), Bayesian information criteria (BIC), entropy, and log-likelihood value. In addition to these indices, interpretability, and parsimony of a model could help in the selection of the final model^15,16^.

Four indicator variables were used for the subgrouping workers. These variables were personal causes of the accident (four categories), managerial causes of the accident (three categories), the severity of accident (three categories), and injured limb (four categories). After identifying the optimal model (four-class model), an LCA was performed with covariates to detect the effect of predictors of latent class membership^16^. To this end, four variables were included in the analysis including age, marital status, education, and shift work. It should be noted that the “critical due to distractions and lack of supervision” class was considered as the reference class when investigating predictors of class membership.

Descriptive statistical analyses were performed using SPSS 16. The LCA was performed using PROC LCA in SAS 9.2 (*P*-value < 0.05).

### Ethical approval

The study was approved by the Ethical Committee of Ardabil University of Medical Sciences, Iran (Code of ethics: IR. ARUMS. REC.1398.073).

### Consent to participate

The informed consent was waived by the Institutional Review Board of Ardabil University of Medical Sciences (Ethical ID: IR. ARUMS. REC.1398.073).

The authors confirm that all methods were carried out in accordance with relevant guidelines and regulations.

### Consent to publish

All the authors agreed to publish the data in this journal.

## Result and discussion

The demographic characteristics of the study participants are given in Table 1. According to Table 1, the most common causes of OA were distractions and lack of adequate and accurate supervision. Also, OA has mainly caused disability in workers, and the eyes, head, face, and neck have suffered the most damage.

**Table 1:** Demographic characteristics, occupational accident type, reason, and severity.

Items	Total (290)
	*N* (%)
Age, Mean (SD)	34.96 ± 10.01
**Gender**	
Male	290(100)
Female	0(0)
**Marital status**	
Unmarried	78(26.9)
Married	212(73.1)
**Education**	
Illiterate	79(27.2)
High school diploma	194(66.9)
Academic	17(5.9)
**Shift work**	
Morning	223(76.9)
Evening	52(17.9)
Night	15(5.2)
**Individual causes of the accident**	
Lack of skills and experience	58(20.0)
Fatigue and excessive sleepiness	57(19.7)
Distractions	83(28.6)
Lack of safety knowledge	92(31.7)
**Managerial causes of the accident**	
Lack of adequate and accurate supervision	121(41.7)
Wrong order	101(34.8)
Lack of occupational safety and health training	68(23.4)
**Severity of accident**	
Death	61(21.2)
Not disabled	40(13.8)
Disabled	189(65.2)
**Injured member**	
Eyes, head, face and neck	95(32.8)
Waist	49(16.9)
Arm, forearm, wrist and fingers	82(28.3)
Feet, knees, and toes	64(22.1)

Table 2 lists different measures of model selection for classes one to six. The number of parameters was relatively high in this study, which could be related to the large categories of indicator variables. When there are numerous parameters/categories involved, the distribution of the G2 reference statistics is unknown. Therefore, no P-value is reported for testing the efficiency of the models. Under these circumstances, AIC and BIC have a more highlighted role in the selection of the best model. According to Table 2, the lowest BIC value was found for the two-class model and the lowest value of AIC value was found for the four-class model. Considering these criteria and the interpretability of the results, the four-class model was chosen for the subgrouping of the workers.

**Table 2:** Comparison of LCA Models with different latent classes based on model selection Statistics.

Number of latent class	Number of parameters estimated	G^2^	df	AIC	BIC	Entropy	Maximum log-likelihood
1	10	270.93	133	290.93	327.63	–	− 1355.14
2	21	173.71	122	215.71	292.78	0.82	− 1306.53
3	32	113.29	111	177.29	294.73	0.76	− 1276.33
**4**	**43**	**87.03**	**100**	**173.03**	**330.83**	**0.75**	** − 1263.19**
5	54	71.14	89	179.14	377.31	0.73	− 1255.25
6	65	61.04	78	191.04	429.58	0.78	− 1250.20

*LCA* latent class analysis, *AIC* Akaike information criterion, *BIC* Bayesian information criterion.

Significant values are in [bold].

Table 3 represents the four-class latent model. Industry workers in our study were grouped into “critical due to distractions and lack of supervision” class (40.1%), “critical due to lack of safety knowledge” class (27.9%), “critical due to fatigue and lack of supervision” class (13.1%), and “catastrophic” class (18.8%). Workers in the “critical due to distractions and lack of supervision” class had a high probability of being distracted and having an accident.

**Table 3:** The four latent class models of occupational accidents in industry workers.

	Latent class			
	Critical due to distractions and lack of supervision	Critical due to lack of safety knowledge	Critical due to fatigue and lack of supervision	Catastrophic
Latent class prevalence	0.401	0.279	0.131	0.188
**Item-response probabilities**				
**Individual causes of the accident**				
Lack of skills and experience	0.284	0.106	0.328	0.071
Fatigue and excessive sleepiness	0.113	0.074	0.652	0.237
Distractions	0.570	0.025	0.013	0.259
Lack of safety knowledge	0.033	0.795	0.007	0.434
**Managerial causes of the accident**				
Lack of adequate and accurate supervision	0.679	0.070	0.574	0.263
Wrong order	0.223	0.462	0.423	0.393
Lack of occupational safety and health training	0.097	0.468	0.002	0.343
**Severity of accident**				
Death	0.002	0.001	0.334	0.875
Non- disabled	0.213	0.132	0.002	0.081
Disabled	0.785	0.866	0.664	0.044
**Injured member**				
Eyes, head, face and neck	0.188	0.169	0.230	0.926
Waist	0.144	0.150	0.450	0.053
Arm, forearm, wrist and fingers	0.323	0.401	0.313	0.002
Feet, knees, and toes	0.344	0.280	0.007	0.019

The probability of a “No” response can be calculated by subtracting the item-response probabilities shown above from 1.

*Item-response probabilities > .5 in bold to facilitate interpretation.

In this class, among managerial causes of the accident, lack of adequate and accurate supervision had the highest probability. Workers in the “critical due to lack safety knowledge” class had a high probability of lacking safety knowledge and becoming disabled. Workers in the “critical due to fatigue and lack of supervision” class had a high probability of being fatigued, having excessive sleepiness, and becoming disabled. In this class, among managerial causes of the accident, lack of adequate and accurate supervision had a higher probability. Finally, workers in the “catastrophic” class had a high probability of death. In this class, the probability of eyes, head, face, and neck injuries was high.

Only one significant predictor of latent class membership was found (Table 4), implying different distribution of latent class membership across this factor. Being illiterate significantly increased the odds of membership in “critical due to fatigue and lack of supervision” (OR = 4.05) and “catastrophic” (OR = 18.99) classes compared to “Critical due to distractions and lack of supervision” classes. Therefore, the level of education is effective in the occurrence of OA.

**Table 4:** Predictors of membership in latent classes of occupational accidents among industry workers.

Predictors	Critical due to lack of safety knowledge	Critical due to fatigue and lack of supervision	Catastrophic	*P*-value
	OR(95%CI)	OR(95%CI)	OR(95%CI)	
Age	0.99(0.95–1.03)	0.99(0.94–1.04)	0.94(0.89–0.98)	0.0774
Marital status (being single)	1.10(0.53–2.29)	0.53(0.14–1.92)	1.42(0.57–3.53)	0.7366
Education (illiterate)	1.34(0.62–2.90)	4.05(1.50–10.94)	18.99(8.08–44.65)	< 0.001
Shift work (night)	0.51(0.17–1.52)	0.81(0.20–3.23)	0.55(0.13–2.32)	0.8218

In this study, we evaluated the pattern of OA among industry workers with the LCA approach. We were able to identify four distinct classes of OA named as critical to distractions and lack of supervision, critical due to lack of safety knowledge, critical due to fatigue and lack of supervision, and catastrophic that represented 40.1%, 27.9%, 13.1%, and 18.8% of the workers in our study sample, respectively.

To the best of our knowledge, there are only a few studies that have employed the LCA approach to detect the latent classes of OA. Moreover, researchers have used various variables to subgroup workers. Some of these studies are discussed below:

Nowakowska and Pajęcki applied LCA to identify OA patterns. They found three severe accident patterns and two light accident patterns^17^. Farnia et al. used LCA to find causation in occupational fatalities in Italy. The authors selected the eight-class model. In their study, most of the factors fell in the class “fall from height or vehicle rollover due to incorrect practice^18^. Although the number of classes was different in the mentioned studies in comparison to our findings, however, inconsistent with other studies our study demonstrated that some workers fell under catastrophic class with a high probability of death. OA usually causes severe or even lethal damage to individuals. They also create large losses to society and the economy. Detecting the mechanisms that frequently cause such accidents can help us develop efficient tools to improve work safety^17,19^.

The present study represented that among the individual factors of accidents, lack of safety knowledge was the main cause. This personal factor had a high probability in latent class 2 (Critical due to lack of safety knowledge). Also, fatigue and excessive sleepiness, and distractions had a high probability of occurring in latent classes 3 and 1 respectively. However, among individual factors, lack of skills and experience had no important role in the clustering of workers. Therefore, in order to prevent OA, it is necessary to pay more attention to fatigue and excessive sleepiness, distractions, and lack of safety knowledge. Zhang et al. showed in their study on the main causes of accidents that the key factors in lack of safety culture were negligence of safety codes and regulations, negligence in paying attention to safety priorities, limited participation in operational parts, and not paying attention to safety education. Therefore emphasized the role of departments, safety communications, safety participation, and supervision of safety culture improvement measures to decrease OA^20^.

Kelly et al. analyzed human factors in accidents and showed that workers experienced distraction and fatigue at work. Therefore, human factors are the main elements of accidents. The common factors in decision making and skill-based errors and communicational errors were coordinating and programming. Increasing awareness of activities and specific occupational situation, educations focused on better decision-making and revising basic skills were essential for preventing accidents^21^.

Baby et al. examined the role of individual factors and safety atmosphere in OA and showed that these factors along with workers’ health conditions were significantly related to OA. Individual factors including age, type of job, education, and experience had a notable effect on the safety behavior of workers. All the personal factors had a notable effect on the safety atmosphere. The study highlighted the need for safety participation, safety knowledge, safety education, and intervention to decrease personal problems at work^22^.

Our findings indicated that most of the workers suffer from an OA disability (Table 1). The most commonly injured limbs in the present study were the eyes, head, face, and neck. Izadi et al.^23^ found that less than 1% of OA resulted in death in Iran from 2007 to 2016 and the highest incidence of these OA was seen in the industrial sectors during all years. There are some theories for the occurrence of severe OA. The causes of this OA are different and depend on many factors such as demographic and occupational characteristics, organizational culture, psychosocial and economic factors, etc. from an economic view, in addition to disability, many workdays are lost in OA. But many countries especially developing ones often encounter with lack of resources to maintain an effective safety management system.

As the results showed, lack of adequate and accurate supervision was the main factor among managerial factors of OA. Therefore, periodic monitoring and improving the quality of monitoring can be helpful in preventing accidents.

Study Tau et al. found that the human factor had a role in 71% of accidents. Among the human factors, the main factor was “lack of supervision”, which had a direct and indirect effect on the recorded accidents. Implemented procedures for safety management were mostly inefficient because of poor supervision. Supervision can affect safety culture as a supervisor has a notable role in the improvement of work methods and processes and the removal of system weak spots. Inefficient supervision is an indicative of inefficient safety culture, which can increase the rate of accidents^24^.

Also, Soltanzadeh et al. showed in their study on the causal factors in the severity of OA that personal and organizational factors, HSE educational factors, and risk management systems had a significant relationship with the severity of the accidents^25^.

## Conclusion

Workers in the “critical due to distractions and lack of supervision” class had a high probability of being distracted and having an accident. And in the “critical due to fatigue and lack of supervision” the class had a high probability of being fatigued, having excessive sleepiness, and becoming disabled. In these two classes, among managerial causes of the OA, lack of adequate and accurate supervision had a higher probability. Workers in the “critical due to lack safety knowledge” class had a high probability of lacking safety knowledge and becoming disabled. And in the “catastrophic” class had a high probability of death. In this class, the probability of eyes, head, face, and neck injuries was high. Among the individual factors of OA, lack of safety knowledge was the main cause. Also, fatigue and excessive sleepiness, and distractions had a high probability of occurring. In order to prevent OA, it is necessary to pay more attention to fatigue and excessive sleepiness, distractions, and lack of safety knowledge. Lack of adequate and accurate supervision was the main factor among managerial factors of OA. Therefore, periodic monitoring and improving the quality of monitoring can be helpful in preventing OA.

## Data Availability

The data used and analyzed during the current study are available from the corresponding author upon reasonable request.
